# Flavonoids and Insulin-Resistance: From Molecular Evidences to Clinical Trials

**DOI:** 10.3390/ijms20092061

**Published:** 2019-04-26

**Authors:** Benedetta Russo, Fabiana Picconi, Ilaria Malandrucco, Simona Frontoni

**Affiliations:** 1 Unit of Endocrinology, Diabetes and Metabolism, S.Giovanni Calibita, Fatebenefratelli Hospital, 00186 Rome, Italy; benedetta_russo6@msn.com (B.R.); fabipicco@gmail.com (F.P.); ilariamalandrucco@outlook.com (I.M.); 2 Department of Systems Medicine, University of Rome Tor Vergata, 00133 Rome, Italy

**Keywords:** metabolic syndrome, insulin-resistance, flavonoids

## Abstract

Insulin-resistance is one of the main factors responsible for the onset and progression of Metabolic Syndrome (MetS). Among all polyphenols, the effects of flavonoids and their main food sources on insulin sensitivity have been widely evaluated in molecular and clinical studies. The aim of this review is to analyse the data observed in vitro, in vivo and in clinical trials concerning the effects of flavonoids on insulin resistance and to determine the molecular mechanisms with which flavonoids interact with insulin signaling.

## 1. Introduction

Metabolic syndrome (MetS) is a clustering of several metabolic abnormalities including abdominal obesity, insulin resistance, hypertension and dyslipidemia [1]. Its presence has been associated with an increased risk of type 2 diabetes mellitus (T2DM) [2] and cardiovascular disease (CVD) [3]. The etiology of MetS is multifactorial as it derives from a complex interaction between genetic, metabolic and environmental factors [4]. According to the new definition of International Diabetes Federation (IDF), a person suffering from MetS has central obesity defined as circumference waist ≥94 cm for Europid men and ≥80 cm for Europid women, with specific ethnicity values for other groups, plus any two of the following four factors: raised trygliceride (TG) level (>150 mg/dL or specific treatment for this lipid abnormality), reduced high density lipoprotein (HDL) cholesterol (men <40 mg/dL, women <50 mg/dL or specific treatment for this lipid abnormality), raised blood pressure (systolic BP >130 mmHg or diastolic BP >85 mmHg or treatment of previously diagnosed hypertension), and raised fasting plasma glucose (>100 mg/dL or previously diagnosed T2DM) [5] (Table 1).

Worldwide prevalence of MetS ranges from 10 to 84% depending on ethnicity, age, gender, and race of the population [3]. As an average, one quarter of the world’s adult population is estimated to have MetS [6].

A number of leading chronic diseases have been shown to be associated with MetS [7]. These include CVD [8], T2DM [9], chronic kidney disease (CKD) [10], non-alcoholic fatty liver (NAFLD) disease [11] and polycystic ovary syndrome (PCOS) [12]. MetS and its comorbidities are associated with premature mortality, therefore it represents a public health problem worldwide [1].

The pathogenesis of MetS is insulin resistance [13], and it is closely related to abdominal obesity [14]. As a consequence, the most appropriate approach to the treatment of MetS should focus on weight loss obtained by lifestyle changes, including diet and physical activity [15]. In particular, the Mediterranean diet which includes fruit, vegetables, legumes, nuts and olive oil has been recognized as the most effective dietary pattern in the prevention and progression of MetS [16]. Its beneficial effects are related to the high content of bioactive compounds, monounsatured and polyunsatured fatty acids and polyphenols [17].

Polyphenols are a large and heterogeneous group of phytochemical compounds of plant origin and are divided into flavonoids, phenolic acids, stilbenes and lignans. It has been shown that polyphenols have anti-oxidant and anti-inflammatory properties [18] and that their beneficial effects in the onset and progression of MetS are mediated by body weight and blood pressure reduction, and improvement in insulin-sensitivity and lipid metabolism [19]. Among all polyphenols, flavonoids are the most abundant in the Mediterranean diet [20] and their well-recognised anti-oxidant and anti-inflammatory properties are of interest for the potential role in the prevention of T2DM [21], that has been shown by recent evidences [22]. With regard to these findings, numerous molecular and clinical studies have been conducted to evaluate the effect of flavonoids on insulin resistance [21].

This review summarizes the results of molecular and clinical studies that evaluated the effects of flavonoids and their major food source on insulin sensitivity, which plays a relevant role in the physiopathogenesis of MetS.

## 2. Flavonoids

Flavonoids are a class of natural products; particularly, they belong to a class of plant secondary metabolites having a polyphenolic structure. In plants, they have several functions, such as providing protection against harmful UV radiation and plant pigmentation. They represent a large group of polyphenolic compounds widely found in fruits, vegetables, whole grains and legumes as well as in cocoa, tea, coffee, nuts, olive oil and red wine [23].

They are characterized by the basic C6-C3-C6 skeleton and two benzene rings. The two aromatic rings within the flavonoid basic chemical structure are linked by a heterocyclic ring, which differs in the degree of oxidation. Hydroxyl group substituents provide centres for reaction [24]. Flavonoids can be divided into different sub-classes based on their chemical structure: flavones, flavonols, flavanols, flavanones, isoflavones and anthocyanins (Table 2). The biochemical actions of flavonoids depend on the presence and position of various hydroxyl substituent groups [23]. Table 2 shows sub-classes and sub-types of flavonoids and their main food source.

A number of clinical and research studies suggest that flavonoids have positive effects in the prevention of several diseases, such as T2DM and CVD [25]. Over the years, scientific evidences indicated that the intake of flavonoids and their major food source may exert anti-oxidant and anti-inflammatory effects [23]. Consequently, flavonoid-rich foods, beverages and extracts, as well as pure flavonoids are studied for the prevention and/or improvement of MetS and MetS comorbidities [20]. The data of these studies suggest that flavonoids may have an effect on insulin sensitivity.

## 3. Insulin Signaling in Normal and Impaired Insulin-Sensitivity

As it is known, insulin acts by binding to its receptors on the membrane of target cells. Following the interaction with its receptors, insulin induces a glucose uptake in skeletal muscle and adipose tissues whereas in the liver it decreases the glucose production and output [26].

**Figure 1:**
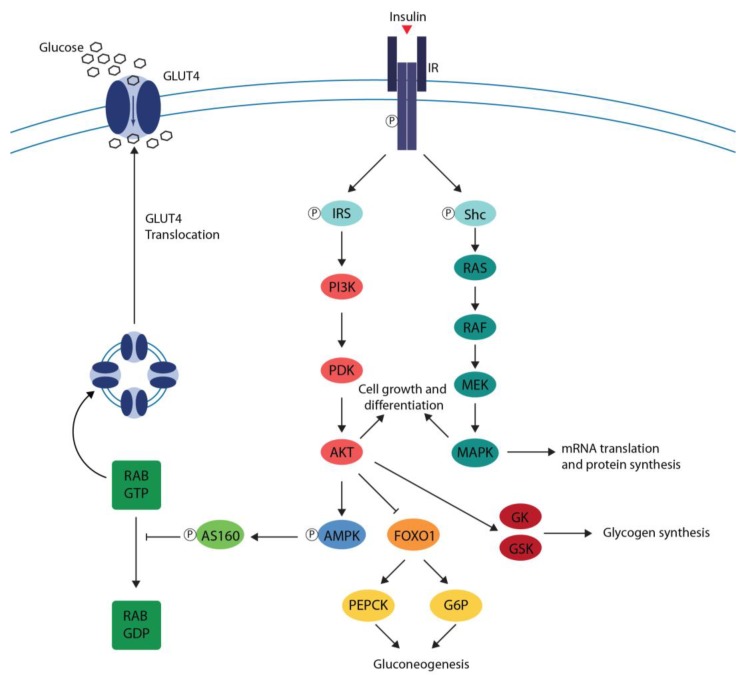
Normal insulin signaling. Insulin binds to the insulin receptor (IR) inducing a conformational change and a rapid autophosphorylation of IR leading to the recruitment and phosphorylation of receptor substrates such as insulin receptor substrate (IRS) and Shc proteins. Shc proteins activate the Ras/mitogen-activated protein kinase (MAPK), whereas IRS proteins mostly activates the phosphoinositide 3-kinase (PI3K)/Akt pathway by recruiting and activating PI3K. In the skeletal muscle and adipose tissue, the PI3K/Akt pathway induces AMP-activated protein kinase (AMPK) phosphorylation and the expression of the glucose transporter type 4 (GLUT4) and its translocation from intracellular vesicles to the cell membrane promoting the uptake of glucose. In the liver, the PI3K/Akt pathway inhibits the expression of phosphoenol pyruvate carboxykinase (PEPCK), and glucose-6-phosphatase (G6P) suppresses gluconeogenesis and activates glucokinase (GK) and glycogen synthase kinase (GSK), promoting glycogen synthesis. Arrow stimulates, T bar inhibits

The interaction of insulin with insulin receptor (IR) induces a conformational change and a rapid autophosphorylation of IR leading to the recruitment and phosphorylation of receptor substrates such as insulin receptor substrate (IRS) and Shc proteins. Shc proteins activate the Ras/mitogen activated protein kinase (MAPK) pathway, whereas IRS proteins mostly activate the phosphoinositide 3-kinase (PI3K)/Akt pathway by recruiting and activating PI3K. The PI3K/Akt pathway is responsible for most of the metabolic effects of insulin while the Ras/MAPK pathway is involved in the regulation of gene expression and, in cooperation with the PI3K pathway, in the control of cell growth and differentiation [27] (Figure 1).

In the skeletal muscle and adipose tissue, the PI3K/Akt pathway induces AMP-activated protein kinase (AMPK) phosphorylation and the expression of the glucose transporter type 4 (GLUT4) and its translocation from intracellular vesicles to the cell membrane promoting the uptake of glucose. In the liver, PI3K/Akt pathway suppresses gluconeogenesis and promotes glycogen synthesis [26] (Figure 1).

**Figure 2:**
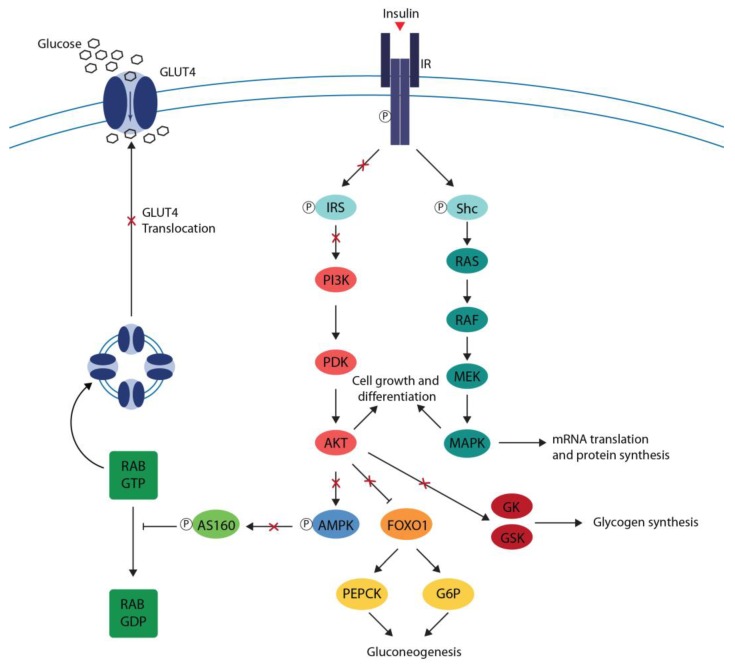
Impaired Insulin signaling. Insulin resistance impairs the activation of PI3K/Akt of the skeletal muscle and adipose tissue leading to a decreased GLUT4 expression and translocation, resulting in impaired glucose uptake. Deficits in hepatic insulin signaling release FOXO1 back to the nucleus to promote the expression of PEPCK and G6P genes promoting gluconeogenesis and decrease GK and GSK activation suppressing glycogen synthesis. Arrow with red X: impaired stimulation, T bar with red X: impaired inhibition

Insulin resistance impairs the phosphorylation signaling pathway of the skeletal muscle and adipose tissue, leading to a decreased GLUT4 expression and translocation resulting in impaired glucose uptake. In the liver, insulin resistance promotes gluconeogenesis and suppresses glycogen synthesis [28] (Figure 2).

It has been demonstrated that the accumulation of adipose tissue at visceral level is associated with a defect in insulin action [29]; visceral adipose tissue releases an excessive amount of free fatty acids (FFA) which interfere with insulin signaling [27]. Moreover insulin resistance is associated with a low degree of inflammation of the adipose tissue and with an increased production and secretion by the latter of a wide range of pro-inflammatory molecules including tumor necrosis factor- α (TNF-α) and interleukin-6 (IL-6), which have systemic effects on peripheral organs interfering in an inhibitory way with the insulin signal [30]. In addition, mutations in IR and in insulin signaling molecules genes have been associated with insulin resistance [27].

## 4. Molecular Mechanisms of Flavonoids and Insulin-Sensitivity

Numerous scientific evidences have shown that polyphenols can interact with several molecular pathways involved in glucose metabolism [31]. Over the years, the effects of several sub-classes of flavonoids on the insulin-signaling pathway have been widely assessed in in vitro experiments (Table 3) and in animal models (Table 4).

### 4.1. Effects of Flavonoids on Insulin-Sensitivity In Vitro

Flavanol extracted from grape seeds has been reported to be an insulinomimetic agent as it stimulates the glucose uptake in 3T3-L1 adipocyte and L6E9 muscle cells via the PI3K/Akt-pathway [32]. Among all flavanols, the effect of Epigallocatechin gallate (EGCG), the most abundant catechin in green tea, has been widely examined in vitro. Experiments conducted on rat L6 skeletal muscle cells showed that EGCG decreases insulin-resistance induced by dexamethasone. A 24h treatment with 20 μM of EGCG inhibits the effect of dexamethasone on insulin sensitivity, improving the glucose uptake. The results showed that EGCG activates the PI3K/Akt pathway and increases the phosphorylation of AMPK, promoting GLUT4 translocation [33]. In addition, a study conducted on insulin-resistant 3T3-L1 adipocyte cells showed that EGCG promotes GLUT4 translocation by improving oxidative stress [34], suggesting that the antioxidant effect of EGCG may improve insulin-signaling transduction; in particular, it was found that only the concentration of 5 μM of EGCG had significantly increased the glucose uptake. The anti-inflammatory effect of EGCG on insulin-sensitivity was also assessed in several studies. The results showed that EGCG reduces the expression of pro-inflammatory adipocytokine resistin in 3T3-L1 adipocyte cells and its effect depends on MAPK pathways, in particular EGCG selectively decreases the amounts of p-ERKs in adipocytes [35]. Additionally, EGCG inhibits TNF-α-induced activation of nuclear factor kappa-light-chain-enhancer of activated B cells (NF-kB) signaling cascade involved in inflammation in 3T3-L1 adipocyte cells [36]. These effects were associated with a decreased insulin resistance [35,36].

The effect of EGCG on the insulin-signaling pathway has also been studied in a H4IIE rats hepatic cells cultures and the results showed a reduced gluconeogenesis and glucose output after exposure to EGCG [37]. A later study assessed the role of EGCG in gluconeogenesis using hepatocytes exposed to a physiologically relevant concentration of EGCG (<1 μM). The data observed suggest that EGCG decreases glucose production by inhibiting the expression of PEPCK and G6P. In addition, this study demonstrated that EGCG activates AMPK and that it exerts toxic effects on primary hepatocytes at a concentration of 10 μM [38], suggesting a dose-dependent effect of EGCG.

The effect of quercetin, the flavonoid belonging to the flavonols group, has also been evaluated. A study conducted on 3T3-L1 adipocyte cells under basal and inflammatory conditions has investigated the effects of quercetin on AMPK activation and GLUT4 translocation. The results in basal conditions showed that quercetin inhibited GLUT4 translocation by inhibiting AS160 phosphorylation. Differently, when the inflammatory challenge impaired insulin action in 3T3-L1 adipocyte cells, quercetin inhibited IκB kinase β (IKKβ) phosphorylation and facilitated insulin signaling leading to the restoration of AS160 phosphorylation and GLUT4 translocation [39].

Furthermore, the effect of quercetin, isolated from berry extract, on glucose uptake was assessed in C2C12 skeletal rat myoblasts. The results showed that the treatment with quercetin increases insulin sensitivity through the activation of the AMPK-signaling pathway [40].

The effect of quercetin on insulin sensitivity has also been investigated in a study conducted on an insulin-resistant NAFLD hepatic cells model induced by FFA. The results suggest that quercetin induces phosphorylation of IR and IRS1, improving insulin resistance [41]. On the contrary, the opposite results were observed in experiments conducted on neuronal cells aimed to evaluate the effect of quercetin on the neuronal control of glucose homeostasis. A study conducted on HT-22 mouse hippocampal neuronal cells has shown that treatment with quercetin inhibits Akt phosphorylation leading to impaired glucose homeostasis [42]. Therefore, these data suggest that quercetin may improve insulin sensitivity in peripheral tissues while at the central neuronal level, quercetin inhibits the insulin-signaling pathway. This discrepancy of the effect of quercetin on peripheral tissues and central neurons is still unclear.

Another flavonol widely examined is kaempferol; studies conducted on rat soleus muscle cells [43] and mature adipocytes [44] showed that kaempferol improves insulin sensitivity by increasing glucose uptake. Experiments conducted on C2C12 myoblast exposed to high-fat acid showed that impaired glucose uptake was reversed by treatment for 24 h with 10 μM of kaempferol [45]. Additionally, a kaempferol treatment (10–20 μM) of 3T3-L1 adipocyte cells activated the insulin transduction pathway by up-regulating the phosphorylation of the IR and IRS1, and increased adiponectin secretion [46]. Contrarily, other authors observed opposite results for 20 μM kaempferol treatment of the same cell line, demonstrating inhibited glucose uptake in differentiated 3T3-L1 adipocyte cells by interfering with the insulin signaling pathway and also by directly interacting with GLUT4 [47]. These data suggest that kaempferol may exert a dose-dependent effect in adipocytes.

Regarding flavanones, it has been shown that naringenin increases L6 rat myotubes glucose uptake in a dose-dependent manner by promoting phosphorylation of AMPK; the maximum stimulation of the glucose uptake was seen with 75 μM of naringenin [48]. Nevertheless, in 3T3-L1 adipocyte cells, naringenin repressed glucose uptake [49]. These data suggest that the effect of naringenin on insulin-mediated glucose uptake might depend on the type of cell.

Experiments were also conducted to assess the effect of blueberry juice on glucose uptake and the results suggest that the juice extract improves insulin-sensitivity by increasing AMPK phosphorylation and consequently promotes glucose uptake in both cultured muscles and adipocyte cells [50]. The flavonoid mostly present in blueberry juice is the anthocyanin. Over the years, the most examined anthocyanin was cyanidin. A recent study showed an enhanced glucose uptake in human skeletal muscle cells after treatment with cyanindin extract from elderberry [51]. The same results were observed in experiments conducted on human and 3T3-L1 adipocyte cells. These experiments showed an improvement of insulin sensitivity by increasing GLUT4 translocation after treatment with cyanidin [52]. Furthermore, a study conducted on H4IIE rat liver cells showed that cyanidin decreases gluconeogenesis by enhancing the down regulation of G6P [53].

The isoflavone, a flavonoid found in soy, is characterized by the estrogenic effect; however, its effects on insulin sensitivity have been evaluated. Isoflavones identified from a branch extract fraction of the Vietnamese traditional herb *Tetracera scandens*, significantly stimulated the glucose uptake both in basal and insulin resistance-stimulated L6 skeletal muscle cells in a dose-dependent manner [54]. A later study conducted on the same cell lines determined the effect of genistein on glucose uptake under normal glucose (5.5 mM) and high glucose (25 mM) conditions [55]. The results showed that genistein dose-dependently and significantly stimulates glucose uptake at concentrations of 10–50 μM under normal glucose conditions. Under the high glucose condition, the maximum increase in glucose uptake was observed at 30 μM of genistein. This study suggested that genistein promotes glucose uptake by inducing AMPK phosphorylation and GLUT4 expression and translocation. The effect of genistein has also been investigated in adipocyte cells under inflammatory conditions. In 3T3-L1 adipocyte cells treated with macrophage-derived conditioned medium (Mac-CM), the GLUT4 translocation was decreased, and it has been shown that 10 μM of genistein restores GLUT4 translocation by activating AMPK [56]. This data suggests that the anti-inflammatory effect of genistein leads to an improvement in insulin signaling. Contrarily, the inhibitory effect on glucose uptake has been observed in 3T3-L1 adipocyte cells with 20–50 μM of genistein [57], suggesting a dose-dependent effect of genistein.

In view of the foregoing, flavonoids may exert direct effects on insulin-signaling pathways, leading to an improvement of insulin sensitivity. The results reported above suggest that flavonoids induce IR and IRS phosphorylation and activate the PI3K/Akt pathway and AMPK promoting GLUT4 expression and translocation in skeletal muscle and adipocyte cells cultures. In addition, experiments conducted on hepatic cells cultures suggest that flavonoids decrease PEPCK and G6P expression, suppressing gluconeogenesis and increasing GK and GSK expression, promoting glycogen synthesis.

### 4.2. Effects of Flavonoids on Insulin-Sensitivity In Vivo

Over the years, scientific evidences have strongly supported the contention that grape seed extract (GSE) improves hyperglycaemia and hyperinsulinemia in high-fructose-fed induced insulin-resistance rats. The results showed that the supplementation of GSE enhanced the expression of Akt, AMPK and GLUT4. Moreover, it has been observed that GSE also increased the mRNA expression of adiponectin. These results suggested that GSE improves the defective insulin-signaling pathway in the skeletal muscle tissue, resulting in improved insulin resistance in fructose-fed rats [58]. In addition, previous studies conducted on high-fat-fed mice showed that GSE flavanols improve hepatic insulin resistance by increasing the activity of GK-promoting glycogen synthesis [59].

Green tea extract (GTE), a major source of flavanols, was reported to regulate the expression of genes involved in insulin-signaling pathways in the muscle tissue of rats with MetS induced with a high-fructose diet. GTE significantly increased the mRNA levels of IRS1 and GLUT4 in the muscle tissue [60]. Moreover, the same study showed that GTE increases the mRNA levels of GSK-promoting glycogen synthesis. Additionally, it has been demonstrated that GTE significantly increases the insulin sensitivity of adipose tissue of high-fructose-fed rats through an increase of glucose uptake by inducing the expression of GLUT4 [61].

EGCG is the prevalent flavanol in green tea. In a study conducted on high-fat-fed mice supplemented with EGCG for 10 weeks, it has been observed that EGCG reduces fasting glucose and insulin levels. The results showed that EGCG decreases hepatic glucose production through the activation of Akt which in turn reduces the expression of gluconeogenic enzymes [62]. Moreover, it has been demonstrated that EGCG improves insulin resistance in NAFLD mice [63].

The effect of quercetin on insulin sensitivity of skeletal muscles was investigated in high-fat high-sucrose-fed mice. Mice were supplemented with quercetin (30 mg/kg) for 6 weeks. The results showed a significant improvement of glycemic control by quercetin treatment [64]. On the contrary, an impaired neuronal control of glucose homeostasis was observed after the treatment with quercetin; a study conducted on djungarian hamsters showed that the oral treatment with quercetin reduces insulin-induced activation of the PI3K pathway in the arcuate nucleus, leading to impaired insulin sensitivity [42]. This data confirmed the discrepancy observed in vitro of the effect of quercetin on peripheral tissues and central neurons. Therefore, further studies are needed to understand the role of quercetin in the central nervous system on the regulation of glucose homeostasis.

Another flavonol widely examined is myricetin which is present in fruits and vegetables; it has been shown that myricetin improves insulin sensitivity by restoring IR, IRS1 and PI3K/Akt phosphorylation and GLUT4 expression and translocation in the soleus muscle tissue of high-fructose-fed rats [65]. Additionally, it has been observed that kaempferol restores AMPK and GLUT4 expression in skeletal muscle and adipose tissues in high-fat diet-induced obese mice [45].

Regarding flavanones, it has been shown that naringin and hesperidin increase GK mRNA expression by promoting glycogen synthesis and decrease the expression of PEPCK and G6Pase, suppressing gluconeogenesis in type 2 diabetic mice [66].

Genistein is the isoflavone mostly found in soybeans with mainly estrogenic effects. However, their effects on insulin sensitivity have been evaluated even in vivo experiments. In a study conducted on high-fructose-fed rats, treatment with genistein improved insulin resistance by restoring homeostatic model assessment for insulin resistance (HOMA-IR) and quantitative insulin sensitivity check index (QUICKI) values [67]. Furthermore, genistein treatment (1 m/kg/day) on high-fat high-fructose-fed mice for 15 days significantly decreases lipid accumulation and increases IR and IRS1 phosphorylation and PI3K/Akt pathway activation, promoting AMPK phosphorylation in mice liver [68]. In a study conducted on normal mice and mice treated with Mac-CM, opposite effects have been observed on insulin sensitivity under normal and inflammatory adipose tissue. The results showed that genistein reduced insulin sensitivity in normal mice by inhibiting the phosphorylation of IRS and IRS1, leading to the inhibition of GLUT4 translocation in adipocytes. Contrarily, in insulin-resistant mice, genistein improved impaired insulin sensitivity induced by inflammatory stimulus by restoring the IRS1 function and increasing AMPK activity, leading to an improvement in GLUT4 translocation [56].

The results obtained in vivo confirmed direct effects of flavonoids observed in vitro on the insulin-signaling pathway. However, in the last few years, it has been shown that flavonoids may also exert indirect effects on impaired insulin-signaling pathways. A study conducted on high-fructose fed rats has demonstrated that myricetin improves the defective post-receptor insulin-signaling pathway, binding the peripheral *μ*-opioid receptor expressed in the insulin target tissues. The treatment with myricetin enhanced the expression of IRS-1, PI3K, Akt and AS160; phosphorylation of Akt and AS160; and GLUT4 translocation in the soleus skeletal muscles [69].

## 5. Effects of Flavonoids on Beta Cell Survival and Insulin Secretion

As described in in vitro and in vivo studies, flavonoid sub-classes regulate glucose homeostasis interacting with insulin sensitivity. However, there is increasing evidence highlighting the role of flavonoids on beta cell survival and insulin secretion. Studies conducted on insulin-releasing cell lines, isolated pancreatic islets and diabetic animal models, suggest that flavonoid sub-classes may preserve and restore beta cells mass and function [70]. Several studies reported that flavonoids may protect beta cells by an antioxidant effect; in particular, it has been shown that quercetin, epicatechin and naringenin decrease the reactive oxygen species (ROS) level and lipid peroxidation in beta cells protecting them against apoptosis [71,72,73]. In addition, it has been demonstrated that kaempferol preserves the survival of beta cells in high-glucose conditions by enhancing B-cell lymphoma 2 (Bcl-2) expression and reducing caspase-3 level [74]. The anti-inflammatory effect of flavonoids and their association with the integrity of beta cells was also assessed; the results suggest that flavonoids protect beta cells from cytokines by activating the PI3K/Akt pathway [75] and suppressing NF-kB translocation [76]. 

Recent evidences reported that flavonoids may trigger and amplify the pathway of insulin secretion of beta cells [70]. It has been shown that genistein and kaempferol activate the cyclic adenosine monophosphate (cAMP)/protein kinase A (PKA) and phospholipase C (PLC)/protein kinase C (PKC) pathway, enhancing glucose-stimulated insulin secretion from beta cells [74,77]. Moreover, a study conducted on INS-1 cell line and rat isolated pancreatic islets suggests that quercetin stimulates insulin secretion by increasing Ca^2+^ influx through an interaction with L-type Ca^2+^ channels [78].

## 6. Dietary Flavonoids and Insulin-Sensitivity in Clinical Studies

Results obtained in in vitro and in vivo studies suggested that flavonoids improve insulin-sensitivity and therefore may exert a beneficial effect on insulin-resistance. Subsequently, numerous clinical studies have assessed the effects of flavonoid-rich food intake on insulin resistance (Table 5).

### 6.1. Effects of Anthocyanin Food Sources on Insulin-Sensitivity

In a double-blinded 6-week clinical trial conducted on 32 non-diabetic obese and insulin-resistant subjects, it was shown that the daily intake of smoothies with added blueberries, equivalent to 668 mg/day of anthocyanin, improved insulin sensitivity measured by a hyperinsulinemic-euglycemic clamp (*p* = 0.04), which represents a gold-standard method to assess insulin sensitivity. This result did not occur as a result of daily intake of smoothies without added blueberry [79]. Furthermore, beneficial effects of anthocyanin from bilberry and black currant on improving insulin resistance were also observed in a 12-week randomized doubled-blinded placebo-controlled pilot trial conducted in 74 patients with NAFLD [80]. The oral glucose tolerance test indicated that 12-week bilberry and black currant supplementation, containing 320 mg of anthocyanins, significantly decrease the 2-hour loading glucose level compared to control (−18.7% vs −3.8%, *p* = 0.02).

Additionally, in a cross-sectional study of 1997 women aged between 18 and 76 years with an average BMI of 25.2 ± 4.5 intake of flavonoids was calculated from food frequency questionnaires using USDA (U.S. Department of Agriculture) database. Higher anthocyanin-rich food intake was associated with a significantly lower HOMA-IR index (−0.1, *p* < 0.05) [81].

Even though these clinical studies reported relevant results, clinical trials aiming to evaluate the effects of anthocyanins are relatively sparse.

### 6.2. Effects of Flavanols Food Sources on Insulin-Sensitivity

Among flavonoids, the effects of flavanols and their primary food sources, including chocolate and cocoa, have been most widely evaluated in clinical trials.

In a randomized clinical trial conducted on 49 overweight or obese subjects with insulin-resistance and hypertension, a decreased insulin resistance assessed by HOMA2-IR index (−0,31%, *p* < 0.05) was observed after 12 weeks of high-flavanol cocoa daily intake containing 902 mg of flavanols [82]. This result did not occur after 12 weeks of low-flavanol cocoa daily intake, containing 36 mg flavanols, highlighting the dose-dependent effect of flavanols on insulin sensitivity.

Moreover, a randomized cross-over trial conducted on 19 hypertensive and insulin-resistant subjects showed that 2 weeks of consumption of dark chocolate containing 147 mg of flavanols decreased the HOMA-IR index (*p* < 0.05) and increased the QUICKY index (*p* < 0.05) [83]. In addition, a more recent study showed that acute dark chocolate consumption prior to prolonged exercise enhanced insulin sensitivity compared with chocolate consumption alone [84].

In a meta-analysis of five clinical trials conducted on 1106 participants, including healthy subjects and patients with hypertension, overweight/obesity, insulin-resistance or T2DM, the effect of flavanols-rich cocoa or dark chocolate intake for 2–18 weeks has been examined [85]. The results showed a significant effect of cocoa or dark chocolate intake on improving insulin sensitivity (HOMA-IR: −0.94, 95% CI= −0.59, −1.29; *p* < 0.001). Total flavanols intake from cocoa or dark chocolate ranged from 16.6 mg/day to 1080 mg/day and control group included low-flavonoid cocoa, white chocolate, skim milk and placebo capsules.

Another primary source of flavanols is green tea, which has been extensively examined in short-term clinical trials. Green tea has shown to exert cardioprotective benefits in observational studies; however, a randomized controlled trial conducted on 35 subjects with MetS showed that the daily consumption of green tea beverage containing 110 mg of EGCG for 8 weeks had no significant effect on insulin sensitivity or on biomarkers of inflammation [86]. Moreover, a randomized placebo-controlled trial conducted on 41 obese patients with PCOS treated with green tea capsules containing 540 mg of EGCG for 12 weeks did not report a beneficial effect on their glucose metabolism [87]. However, a randomized controlled trial conducted on 60 insulin-resistant subjects showed that an 8-week daily supplementation of green tea extract powder containing 544 mg of catechins significantly reduced HbA1c (*p* = 0.03) [88]. In a meta-analysis which included 22 randomized clinical trials conducted on 1548 subjects, aged 18 to 70, suffering from overweight/obesity, MetS or TD2M, green tea catechins were shown to have beneficial effects on lowering fasting glucose (−1.48 mg/dL; 95% CI: −2.57, −0.40 mg/dL, *p* = 0.008) but effects on HOMA-IR index were not significant [89]. The daily intake of green tea catechins ranged from 240 to 1207 mg and the results obtained were compared to those associated with water consumption or placebo.

### 6.3. Effects of Isoflavones Food Sources on Insulin-Sensitivity

Recently, some evidence emerged that dietary isoflavones play beneficial roles in metabolic diseases. Considerable attention was focused on the high dietary intake of soy isoflavones [55]. Probably because of these estrogenic properties, most of the clinical trials evaluated the effects of soy isoflavones on postmenopausal or perimenopausal women. In a randomized crossover clinical trial conducted on 42 postmenopausal women with MetS, it was shown that 8-week intake of soy-nut containing 102 mg of isoflavones decreased the HOMA-IR index compared with control diets (−12.9 ± 0.9; *p* < 0.01) [90]. Interestingly, in a meta-analysis that only considered clinical trials conducted among 794 non-Asian perimenopausal or postmenopausal women, soy genistein supplement for 3 months to 2 years significantly reduced serum insulin and HOMA-IR index, but had no effects on fasting blood glucose [91]. The daily intake of genistein ranged from 54 to 120 mg. Additionally, a meta-analysis of 24 clinical trials conducted in 1518 overweight, obese or T2DM men and women did not show significant effects on HOMA-IR index, HbA1c, fasting and 2 h blood glucose or fasting and 2 h insulin levels during oral glucose tolerance test. The intervention groups included soy intake and isoflavones content which ranged from 36 to 132 mg/day [92].

## 7. Conclusions

The studies reported in this review suggest that several sub-classes of flavonoids may improve insulin sensitivity.

**Figure 3:**
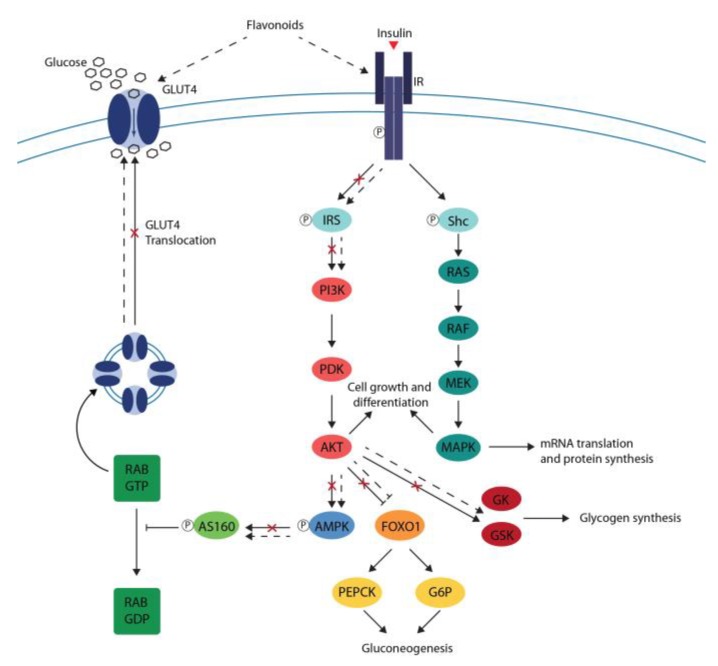
Effects of flavonoids on impaired insulin signaling. Flavonoids induce IR and IRS phosphorylation and activate PI3K/Akt pathway and AMPK, promoting GLUT4 translocation in skeletal muscle and adipose tissues. In the liver, the PI3K/Akt pathway activated by flavonoids decreases PEPCK and G6P expression, suppressing gluconeogenesis and increasing GK and GSK expression, promoting glycogen synthesis.

In particular, studies conducted in vitro and in vivo showed that flavanols, flavonols, flavanones, anthocyanins and isoflavones as well as their main food sources induce glucose uptake in skeletal and adipose tissues and decrease hepatic glucose production and output. These data demonstrated that flavonoid mostly activates the PI3K/Akt pathway in a dose-dependent manner promoting GLUT4 translocation, suppressing gluconeogenesis and stimulating glycogen synthesis (Figure 3). Moreover, the positive effect of flavonoids on insulin-signaling pathways are supported by data observed in clinical trials, as they suggest that the intake of flavonoid-rich food may decrease insulin resistance.

Despite these relevant results, not all molecular and clinical studies demonstrated concordant results. The differences in methodologies, concentrations of flavonoids and observed populations may have contributed to the observation of discording results. The very heterogeneous amounts of flavonoid sub-classes used in molecular and clinical studies do not allow to recommend the level of flavonoids necessary to achieve an improvement of insulin resistance. Further studies are needed to identify the optimal concentration of flavonoids that may exert beneficial effects on insulin sensitivity. Additional clinical studies are also required to confirm the correlation between the intake of each flavonoid sub-class and reduced insulin resistance.

## Figures and Tables

**Table 1:** IDF Metabolic syndrome definition.

Circumference Waist ≥94 cm for Europid Men and ≥80 cm for Europid Women + Two of the Following Four Factors	
Hypertriglyceridemia	>150 mg/dL or specific treatment for this lipid abnormality
Low HDL cholesterol	<40 mg/dL for men or specific treatment for this lipid abnormality <50 mg/dL for woman or specific treatment for this lipid abnormality
Hypertension	>130/85 mmHg or treatment of previously diagnosed hypertension
Raised fasting plasma glucose	>100 mg/dL or previously diagnosed T2DM

**Table 2:** Sub-classes and sub-types of flavonoids and their main food source.

Flavonoid	Structure	Sub-Type	Main Food Source
Flavone	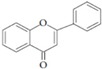	Apigenin, Luteolin, Tangeritin	Virgin olive oil, oranges, whole grain, black olives
Flavonol	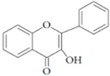	Quercetin, Kaempferol, Myricetin	Spinach, beans, onions, shallot, berry
Flavanol	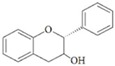	Epicatechin gallate (ECG), catechin, epicatechin	Green tea, grape seed, cocoa, dark chocolate, nuts
Flavanone	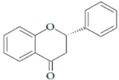	Naringenin, Hesperidin, Naringin	Grape fruit juice, orange juice
Isoflavone	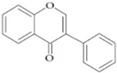	Genistein, Daidzein	Soy
Anthocyanin	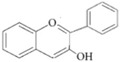	Cyanidin, Apigeninidin	Blueberries, black elderberry, black currant, cherries, red wine

**Table 3:** Effects of flavonoids on insulin sensitivity in in vitro experiments.

Flavonoids	Quantity	Vitro Model	Activity	Effect	Ref.
EGCG	20 μM	L6 rat skeletal muscle cells	Induced phosphorylation of AMPK promoting GLUT4 translocation	Increased glucose uptake	[33]
	5 μM	3T3-L1 adipocyte cells	Improvement of oxidative stress promoting GLUT4 translocation	Increased glucose uptake	[34]
	20–100 μM	3T3-L1 adipocyte cells	Reduced expression of resistin by decreasing the amounts of p-ERKs	Increased glucose uptake	[35]
	0.5–10 μM	3T3-L1 adipocyte cells	Inhibited TNF-α-induced activation of NF-kB signaling cascade	Decreased inflammation	[36]
	25 μM	H4IIE rats hepatic cells	Inhibited expression of PEPCK and G6P	Decreased gluconeogenesis and glucose output	[37]
	1 μM	C57BL/6 rats hepatic cells	Inhibited expression of PEPCK and G6P	Decreased gluconeogenesis and glucose output	[38]
Quercetin	10 μM	3T3-L1 adipocyte cells	Inhibited GLUT4 translocation by inhibiting AS160 phosphorylation in basal condition	Inhibited glucose uptake	[39]
			Inhibited IKKβ phosphorylation and restored AS160 phosphorylation promoting GLUT4 translocation in inflammatory condition	Decreased inflammation and increased glucose uptake	
	25–100 μM	C2C12 skeletal rat myoblasts	Activated AMPK signaling pathway	Increased glucose uptake	[40]
	0.1–100 μM	HepG2 hepatic cells	Induced phosphorylation of IR and IRS1	Enhanced insulin signaling transduction	[41]
	25–100 μM	HT-22 mouse hippocampal neuronal cells	Inhibited Akt phosphorylation	Impaired glucose homeostasis	[42]
Kaempferol	10 μM	C2C12 mouse myoblasts	Induced GLUT4 expression and AMPK activity	Increased glucose uptake	[45]
	10–20 μM	3T3-L1 adipocyte cells	Induced phosphorylation of IR and IRS1	Increased glucose uptake	[46]
			Induced adiponectin secretion	Decreased inflammation	
	20 μM	3T3-L1 adipocyte cells	Inhibited insulin signaling pathway and GLUT4 translocation	Inhibited glucose uptake	[47]
Narigenin	50–150 μM	L6 rat myotubes	Induced phosphorylation of AMPK	Increased glucose uptake	[48]
	6 μM	3T3-L1 adipocyte cells	Inhibited PI3K activity	Repressed glucose uptake	[49]
Cyanidin	0.1–10 μM	Human skeletal muscle cells		Increased glucose uptake	[51]
	50 μmol/L	Human and 3T3-L1 adipocyte cells	Induced GLUT4 translocation	Increased glucose uptake	[52]
	5 μg/mL	H4IIE rat liver cells	Decreased G6P expression	Decreased glucose production	[53]
Genistein	10–50 μM	L6 skeletal muscle cells	Induced PI3K and AMPK phosphorylation and increased GLUT4 expression and translocation	Increased glucose uptake	[54,55]
	10 μM	3T3-L1 adipocyte cells	Induced AMPK phosphorylation and GLUT4 translocation	Increased glucose uptake	[56]
	20–50 μM	3T3-L1 adipocyte cells	Suppressed GLUT4 activity	Inhibited glucose uptake	[57]

**Table 4:** Effects of flavonoids on insulin sensitivity in in vivo experiments.

Flavonoids Source/Flavonoids	Quantity/Day	Animal Models/Length of Study	Activity	Effect	Ref.
GSE	0.5–1% of total diet	High-fructose fed rats/8 weeks	Increased expression of Akt, AMPK, GLUT4 and adiponectin in skeletal muscle tissue	Improvement of insulin resistance in skeletal muscle tissue	[58]
	80 mg/kg	High-fat fed mice/6 weeks	Increased GK activity in hepatic tissue	Improvement of hepatic insulin resistance	[59]
GTE	1–2 g/kg	High-fructose fed rats/6 weeks	Increased mRNA levels of IRS1 and GLUT4 in skeletal muscle tissue	Improvement of insulin resistance in skeletal muscle tissue	[60]
			Increased mRNA levels of GSK in hepatic tissue	Improvements of hepatic insulin resistance	
	0.5 g/100 mL	High-fructose fed rats/12 weeks	Increased expression of GLUT4 in adipose tissue	Improvement of adipose tissue insulin resistance	[61]
EGCG	50 mg/kg	High-fat fed mice/10 weeks	Activation of Akt and decreased expression of PEPCK and G6P in hepatic tissue	Improvement of hepatic insulin resistance	[62]
Quercetin	10 mg/kg	Djungarian Hamsters	Reduced activation of PI3K in arcuate nucleus	Impaired insulin sensitivity	[42]
Myricetin	3 mg/kg	High-fructose fed rats/4 weeks	Restored IR, IRS1 and PI3K/Akt phosphorylation and GLUT4 expression and translocation in soleus muscle tissue	Improvement of insulin resistance in skeletal muscle tissue	[65]
Kaempferol	0.05% of total diet	High-fat fed mice/6 weeks	Increased AMPK and GLUT4 expression in skeletal muscle and adipose tissue	Improvement of insulin resistance in skeletal muscle and adipose tissue	[45]
Naringin/Hesperidin	0.2 g/kg	T2DM mice/5 weeks	Increased expression of GK and decreased PEPCK and G6P expression in hepatic tissue	Improvement of hepatic insulin resistance	[66]
Genistein	1 mg/kg	High-fat high-fructose fed mice/2 weeks	Increased IR, IRS1, PI3K and Akt phosphorylation in hepatic tissue	Improvement of hepatic insulin resistance	[68]
Myricetin	1 mg/kg	High-fructose fed rats/2 weeks	Enhanced expression of IRS-1, PI3K, Akt, AS160, increased Akt and AS160 phosphorylation and Glut4 translocation in soleus skeletal muscles	Improvement of insulin resistance in skeletal muscle tissue	[69]

**Table 5:** Clinical studies evaluating flavonoids sources food intake on insulin sensitivity.

Type of Study	Population/Number of Participants	Length of Study	Flavonoids Food Source	Flavonoids Sub-Class	Quantity Intake	Results	Ref.
Double blinded clinical trial	Obese insulin-resistant/32	6 weeks	Blueberries	Anthocyanin	668 mg/day	Improvement insulin-sensitivity (*p* = 0.04)	[79]
Randomized double blind placebo-controlled pilot trial	NAFLD/74	12 weeks	Bilberry and black currant	Anthocyanin	320 mg/day	Decreased the 2-hour loading glucose level (*p* = 0.02)	[80]
Randomized clinical trial	Overweight or obese insulin-resistant/49	12 weeks	Cocoa	Flavanols	902 mg/day	Decreased insulin- resistance (*p* < 0.05)	[82]
					36 mg/day	No effect on insulin-resistance	
Randomized cross-over trial	Hypertensive and insulin-resistant/19	2 weeks	Dark chocolate	Flavanols	147 mg/day	Decreased insulin-resistance (*p* < 0.05)	[83]
Randomized controlled trial	Mets/35	8 weeks	Green tea	Flavanols	110 mg/day	No effect on insulin-sensitivity	[86]
Randomized placebo-controlled trial	Obese with PCOS/41	12 weeks	Green tea	Flavanols	540 mg/day	No effect on insulin- sensitivity	[87]
Randomized controlled trial	Insulin-resistant/60	8 weeks	Green tea	Flavanols	544 mg/day	Decreased HbA1c (*p* = 0.03)	[88]
Crossover clinical trial	Postmenopausal women with Mets/42	8 weeks	Soy-nut	Isoflavones	102 mg/day	Decreased insulin-resistance (*p* < 0.01)	[90]
